# Temporal Changes in Fecal Unabsorbed Carbohydrates Relative to Perturbations in Gut Microbiome of Neonatal Calves: Emerging of Diarrhea Induced by Extended-Spectrum β-lactamase-Producing Enteroaggregative *Escherichia coli*

**DOI:** 10.3389/fmicb.2022.883090

**Published:** 2022-07-07

**Authors:** Zhiyuan He, Yulin Ma, Xu Chen, Sirui Yang, Shuyuan Zhang, Shuai Liu, Jianxin Xiao, Yajing Wang, Wei Wang, Hongjian Yang, Shengli Li, Zhijun Cao

**Affiliations:** State Key Laboratory of Animal Nutrition, College of Animal Science and Technology, China Agricultural University, Beijing, China

**Keywords:** enteroaggregative *E. coli*, extended-spectrum β-lactamase producing *E. coli*, neonatal dairy calves, unabsorbed carbohydrates, gut microbiome, metabolome

## Abstract

Early gut microbiota development and colonization are crucial for the long-term health and performance of ruminants. However, cognition among these microbiota is still vague, particularly among the neonatal dairy calves. Here, extended-spectrum β-lactamase-producing enteroaggregative *E. coli* (ESBL-EAEC)-induced temporal changes in diversity, stability, and composition of gut microbiota were investigated among the neonatal female calves, with the view of discerning potential biomarkers of this arising diarrhea cases in local pastures. Nearly, 116 newborn calves were enrolled in this time period study during their first 2 weeks of life, and a total of 40 selected fecal samples from corresponding calves were used in this study. The results revealed that differentiated gut microbiome and metabolome discerned from neonatal calves were accompanied by bacterial infections over time. Commensal organisms like *Butyricicoccus, Faecalibacterium, Ruminococcus, Collinsella*, and *Coriobacterium*, as key microbial markers, mainly distinguish “healthy” and “diarrheic” gut microbiome. Random forest machine learning algorithm indicated that enriched fecal carbohydrates, including rhamnose and N-acetyl-D-glucosamine, and abundant short-chain fatty acids (SCFAs) existed in healthy ones. In addition, Spearman correlation results suggested that the presence of *Butyricicoccus, Faecalibacterium, Collinsella*, and *Coriobacterium*, key commensal bacteria of healthy calves, is positively related to high production of unabsorbed carbohydrates, SCFAs, and other prebiotics, and negatively correlated to increased concentrations of lactic acid, hippuric acid, and α-linolenic acid. Our data suggested that ESBL-EAEC-induced diarrhea in female calves could be forecasted by alterations in the gut microbiome and markedly changed unabsorbed carbohydrates in feces during early lives, which might be conducive to conduct early interventions to ameliorate clinical symptoms of diarrhea induced by the rising prevalence of ESBL-EAEC.

## Introduction

Young ruminants are susceptible to diarrhea during their early lives, which is mainly induced by pathogenic *E. coli, Cryptosporidium*, Rotavirus, Coronavirus, and certain remarkable changes in the environmental factors (Cho and Yoon, 2014). Recent developments in the pathogenesis of ESBL-EAEC highlight the disorder of the host immune system, associated with the production of extrinsic toxins, adhesins, and siderophores, and gut microbiota dysbiosis (Chevalier et al., 2021). Indeed, increasing evidence has emphasized the long-time impact of early-life gut microbiome structures on the gut health status of adults (Kerr et al., 2015). Besides, efficient blockage of bacterial adhesion to the surface of intestinal epithelial cells is an effective strategy to control the rising ESBL-EAEC infection cases (Boll et al., 2020). Unfortunately, antimicrobials are still widely used for both prevention and treatment of common infectious pathogens, such as EAEC, in food-producing animals during their early lives (Mathew et al., 2007; Yang et al., 2017), which consequently induce perturbations in the diversity of gut microbiota and produce long-term adverse impacts, including rising susceptibility to pathogens, immunological defects, and incidence of multi-drug resistance (MDR) (Zeissig and Blumberg, 2014; Bakkeren et al., 2019). Indeed, the consumption of rational antibiotics is a powerful tool for disease control; however, the overuse of antibiotics is more cost-effective (Cho and Yoon, 2014). Thus, tracking the diarrheic onset following the changes in the gut microbiome supports early interventions in dairy calves, thus leading to a cut down in antibiotic usage. Previous findings based on neonatal dairy veal calves have predicted temporal changes in the gut microbiota of diarrheal calves (Ma et al., 2020). Therefore, we deduced that the composition of the early-life gut microbiome in these female neonatal calves could reflect the intestinal health status and thus indicate the onset of diarrhea induced by ESBL-EAEC infection.

Actually, temporal changes in the gut metabolome are correlated to perturbations in the gut microbiome of neonatal calves, such as the production of SCFAs and unabsorbed carbohydrates. The SCFAs are mainly produced by colonic bacterial fermentation of unabsorbed carbohydrates and dietary fiber (Cummings et al., 1987). The total SCFA concentrations in the hindgut are significantly different depending on the calf age, considering that total SCFA production increases from day 7 onward, with the highest concentration observed on day 21 (Song et al., 2018). Different fermentation substrates produce different types of SCFAs, and the unabsorbed sugar L-rhamnose mostly yields propionate (Darzi et al., 2016). Actually, L-rhamnose is a natural monosaccharide that is widely found in foods, such as carrots, cabbage, and oranges (Cummings and Englyst, 1987). It cannot be absorbed in the small intestine until it reaches the colon in humans and animals (Byrne et al., 2018). Interestingly, fermentation of L-rhamnose by the rumen bacteria significantly reduces the rate of methanogenesis and upregulates the production of SCFAs (Reinhardt et al., 2019). Among these bacteria, previous studies have shown that various *Clostridial* species are involved in the fermentation of L-rhamnose (Petit et al., 2013), and specific species are able to produce acetic acid, propionic acid, butyric acid, 1,2-propanediol, and *n*-propanol when L-rhamnose is used as the sole carbon and energy source (Diallo et al., 2019). Besides, it is an important ingredient of surface-associated exopolysaccharide (EPS) in many probiotics, thus mediating the displacement of pathogenic organisms through the competitive occupancy of adhesion sites and stimulation of the immune system (Guglielmetti et al., 2010; Hidalgo-Cantabrana et al., 2012).

Herein, we used a female calf as an animal model to represent young ruminants, and aimed to detect core intestinal flora and fecal metabolites post-ESBL-EAEC infection. We hypothesized that ESBL-EAEC infection could induce perturbations in the gut microbiome and temporal changes in the fecal metabolome, particularly with regard to the relative abundance of unabsorbed carbohydrates. Based on the previous publications and existing data, we inferred that alterations in ceratin specific commensal microbiota and fecal metabolite content could be exploited as potential biomarkers for diarrhea induced by ESBL-EAEC infection. In addition, we tried to shed light on the potential hazards and risks associated with emerging ESBL-EAEC infections in young calves, and the alterations in the gut microbiota and fecal metabolome among healthy and diarrheic calves could be employed as predictive biomarkers for diarrhea induced by ESBL-EAEC infection.

## Materials and Methods

### Bacterial Strains and Bacterial Culture

The identified ESBL-EAEC strain 1587 was isolated from our previous clinical trial and was found to be prevalent in the pasture of this study. Genomic information and antibiotic susceptibility of the strain are presented in Supplementary Tables 2, 3 (He et al., 2021). Except for specific instructions, all isolates from fecal samples were incubated on MacConkey agar plates (Luqiao company, Beijing, China) or Luria-Bertani broth (LB, Qingdao Hope Bio-technology) at 37°C. Antibiotics were obtained from the China Institute of Veterinary Drug Control. All *E. coli* isolates were screened for the phenotypic identification of ESBL producers on MacConkey agar containing cefotaxime (2 mg/L), and further confirmation was made using double-disc synergy testing in accordance with CLSI recommendations. Isolates were considered positive when the clear zone of inhibition produced by ceftazidime plus clavulanic acid or cefotaxime plus clavulanic acid was at least 5 mm larger than their respective single disks (CLSI, 2016).

### Calf Health Status Assessment and Fecal Sample Collection

Neonatal female Holstein dairy calves sourced from conventional cow pasture in north China and free from antibiotic treatments were selected for this time period study. All animals were transferred to the separate calf hutches after birth to avoid direct contact, and health conditions were appraised daily to ensure that they were free from disease, injury, and dehydration. The animal experimental protocols were approved by Beijing Association for Science and Technology (ID no. SYXK, 2016-0008). The calves were fed 4 L of colostrum during post-natal care service. The general appearance, fecal score, and respiratory score were recorded according to the previously published methods (Villot et al., 2019). Briefly, a total of 116 neonatal calves were included in our trial, and they were drived to separate fences in 2 days old after birth. They were bucket-fed with 4.5 L of colostrum daily during the first 2–7 days (phase I) and 5.5 L during 8–14 days (phase II). The milk replacer contained 67% of skim milk powder with 260 g/kg crude protein, 160 g/kg crude fat, 10 g/kg crude ash, and 19.2 MJ/kg metabolizable energy on a dry matter basis (Nutrifeed, IN, Netherlands). All these calves were the offsprings of the same litter where feeding multiparous cows of 2 to 3 parity. Besides, the average birth weight of all these calves was 37 kg with a similar body measurement index (rump height, thurl width, and body trunk index). Calves with diarrhea were identified over the time period and were defined as the “diarrhea” group (fecal score was ≥3 for at least 2 days and positive for only ESBL-EAEC) (Lesmeister and Heinrichs, 2004). The remaining calves of the same time period were classified as the “healthy” group (fecal score was ≤2 for at least 2 days and free from main pathogens) (Supplementary Table 1). No calves were treated with antibiotics during this trial. Appropriate equipment and sterile gloves were used for the collection of rectal fecal samples (~10 g) to prevent cross-contamination. Initially, all fecal samples were subjected to the detection of ESBL-EAEC isolates according to a previous method (He et al., 2021) and were further screened using the culture methods mentioned earlier. The common genes encoding ESBL and EAEC were tested by PCR as described previously with minor modifications and further confirmed by sequencing analysis (Dallenne et al., 2010). Then, the positive and negative samples were further tested for the presence of Rotavirus, Coronavirus, and *Cryptosporidium* antigens using commercial ELISA kits, and all the three negative samples were chosen for further analysis (Feldmann et al., 2019). Finally, 40 fecal samples (positive for ESBL-EAEC and negative for Rotavirus, Coronavirus, and *Cryptosporidium* antigens) were enrolled in phase I (9 healthy: H_1 and 11 diarrheal: D_1) and phase II (11 healthy-H_2 and 9 diarrheal-D_2) trials over the entire study period. All the collected fecal samples were immediately stored at −80°C for gut microbiome and metabolome analyses.

### DNA Extraction, PCR Amplification, and 16S rRNA Gene Sequencing

The respective bacterial genomic DNA was extracted from 40 fecal samples, phase I (9 healthy-H_1 and 11 diarrheal-D_1) and phase II (11 healthy-H_2 and 9 diarrheal-D_2), using QIAamp DNA Isolation Kit (Qiagen, Hilden, Germany). Amplicons of V3-V4 hypervariable regions of the 16S rRNA gene were amplified using optimized primers (338F: 5′-ACTCCTACGGGAGGCAGCAG-3′, 806R: 5′-GGACTACHVGGGTWTCTAAT−3′). The amplicon DNA with an optimal size (~450 bp) was purified on 1.2% agarose gel using a QIAquick PCR purification kit (Qiagen Science, MD). The quality and quantity of purified PCR products were checked using Quant-iT PicoGreen dsDNA Assay Kit (Microplate reader, BioTek, FLx800) to ensure that all DNA concentrations were above 25 ng/μl. For the Illumina Miseq sequencing, the PCR product library was prepared using the TruSeq Nano DNA LT Library Prep Kit (Illumina), following sequencing on the Illumina Miseq platform (2 × 300, pair-end).

### Gut Microbiota Profiling

The paired sequences were denoised, quality-filtered, and merged using the DADA2 plugin (version 3.11) to obtain the amplicon sequence variants (ASVs) feature table (Callahan et al., 2016). Taxonomic classification was performed using q2-feature-classifier (QIIME2 microbiome analysis platform, version 2020.02) (Bolyen et al., 2019). Taxonomy was assigned to filtered ASVs using a pretrained QIIME2-compatible SILVA version 132 database, with 99% identity for the bacteria and representative sequences (Quast et al., 2013). Species diversity was determined using q2-diversity of QIIME2 version 2020.02 (http://www.r-project.org/). Bray–Curtis, Jaccard, unweighted UniFrac, and weight UniFrac outputs were assessed and visualized using unsupervised PCoA analysis to contrast bacterial communities between groups using the “ggplot2” package of the R software (version 3.3.1) (http://www.r-project.org/). Differences between the groups were determined using PERMANOVA, ANOSIM, and PERMDISP, with 999 Monte Carlo permutations in the “vegan” package in R software. Differentially abundant genera were identified by performing linear discriminant analysis (LDA) effect size (LEfSe) after analyzing all features using Kruskal–Wallis test and checking whether all the pairwise comparisons between subclasses within different classes significantly agree with the class level trend using the pairwise Wilcoxon test (http://huttenhower.sph.harvard.edu/galaxy/root?tool_id=lefse_upload) (Segata et al., 2011). Alpha values for the Kruskal–Wallis and pairwise Wilcoxon tests were 0.05. A size-effect threshold of 3 on the logarithmic LDA score and average relative abundances >0.01% were used to differentiate the discrepant taxa. Gene family abundance of gut microbial communities was predicted using PICRUSt analysis according to the 16S rRNA gene composition (Langille et al., 2013). The constructed ASV feature table was converted into the PICRUSt format and normalized to 16S rRNA gene copy number to correct for over- and under-estimation of microbial abundance. For potential functional profiles, the normalized dataset was analyzed using the MetaCyc dataset (https://metacyc.org/) and the KEGG database (https://www.kegg.jp/). The abundance of KEGG Orthology (KO), KEGG enzymes, and pathways was normalized to counts per million reads (CPM) for downstream analysis. To establish the model for predicting diarrhea, random forest algorithms (“Random Forest Classifier” package in QIIME2) were used to identify “healthy” and “diarrheal” microbiota based on the relative abundance of specific bacterial genera. The accuracy and feature importance of specific genera were further analyzed. To minimize the potential over-fitting in the model, the three-fold cross-validation was done till model accuracy was determined for each permutation, and then an overall accuracy was estimated (Yatsunenko et al., 2012).

### Untargeted Metabolomic Analyses

Lyophilized fecal samples (5 mg) of healthy or diarrheal calves were homogenated using zirconium oxide beads for 3 min, and 145 μL of extraction solution (containing 25 μL of water and 120 μL of methanol) was further added to extract the metabolites. The samples were homogenated for another 3 min using a high-throughput tissue disruptor and then centrifuged at 1,800 × *g* for 20 min. The acquired supernatant was transferred to a 96-well plate and mixed with 20 μL of derivative reagents at 30°C for 60 min, following procedures of Eppendorf epMotion Workstation (Eppendorf Inc., Humburg, Germany). The sample was further diluted with 330 μL of ice-cold 50% methanol, stored at −20°C for 20 min, and followed by centrifugation (4,000 × *g* for 30 min at 4°C). The supernatants were subjected to LC-MS analysis.

The extracted metabolites were analyzed using a UPLC-MS/MS system (ACQUITY UPLC-Xevo TQ-S, Waters Corp., Milford, MA, USA). Chromatographic separation was performed using a BEH C18 column (2.1 mm × 100 mm, 1.7 μm, Waters). The desolvation and source temperatures were set at 500 and 150°C, respectively. Mobile phases containing acetonitrile/isopropanol (1:1, 0.1% formic acid) and 0.1% formic acid were used as carried liquids at a constant flow rate of 0.4 mL/min.

The acquired raw data were processed using the MassLynx software (version 4.1, Waters, Milford, MA, USA). Each sample was analyzed by UPLC-MS/MS in both positive and negative ionization modes to acquire metabolite profiles. The order of analysis of all test samples was randomized. The quality control (QC) samples were pooled samples in which both the metabolite composition of the samples and sample matrix were mixed, and then analyzed using the same methods to evaluate the quality and variance of the acquired data. Self-developed platform iMAP (version 1.0, Metabo-Profile, Shanghai, China) was used for further statistical analyses of metabolite profiles based on the differences in concentrations between individuals and groups. Bray–Curtis dissimilarity was assessed and visualized using PCoA. The relative concentrations of the metabolites were presented as a heatmap. To establish the model for predicting diarrhea, random forest algorithms (“Random Forest” package in R) were used to identify “healthy” and “diarrheal” microbiota based on the relative abundance of specific metabolites. The accuracy of the selected bacterial genera was then assessed by calculating the area under the receiver-operating characteristic (AUC) (“roc.curve”package) in R. To further minimize the potential over-fitting in the model, a three-fold cross-validation approach (“trainControl” package in R) was applied (Cawley and Talbot, 2010).

### Statistical Analysis

The distance in the coefficient of variation (CV) with regard to the relative abundance of genera in calf feces and predicted genes on each day of growth between the two groups of calves were assessed using the nonparametric Kruskal–Wallis test, and multiple comparisons were conducted using Mann–Whitney–Wilcoxon U-test. A significant difference was observed following the interpretation and visualization of the results. For metabolome studies, two types of statistical analyses were extensively performed: (1) multivariate statistical analyses, such as principal component analysis (PCA), partial least square discriminant analysis (PLS-DA), orthogonal partial least square discriminant analysis (OPLS-DA), random forest, and so on; and (2) univariate statistical analyses, including the nonparametric Kruskal–Wallis test, student's *t*-test, Mann–Whitney–Wilcoxon U-test, ANOVA, correlation analysis, and so on. Statistical algorithms were adapted from the widely used software packages for statistical analysis in R studio (http://cran.r-project.org/). STAMP software was applied to detect the differentially abundant KEGG pathways with false discovery rate correction. Spearman correlation coefficient analysis was used to analyze the correlation between gut microbiota and metabolites using the “ggplot2” and “pheatmap” packages of R software (version 3.3.1). All data were presented as mean ± SEM values unless otherwise indicated. Statistical significance was determined for ^*^P < 0.05, ^**^P < 0.01, and ^***^P < 0.001.

## Results

### Perturbations in Microbial Diversity and Stability of Gut Microbiota Post-ESBL-EAEC Infection

**Figure 1:**
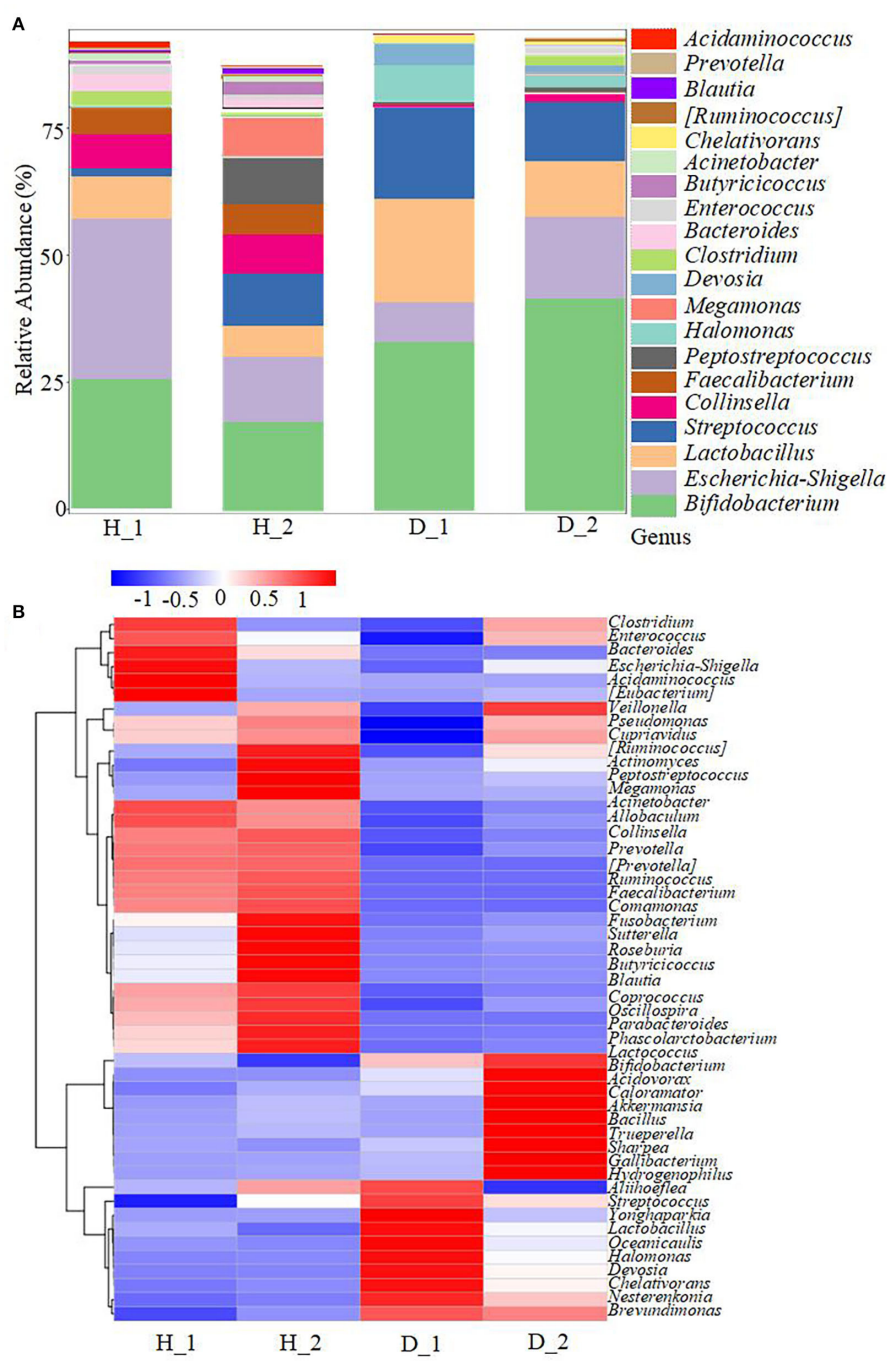
Gut microbiota assembly of neonatal calves post-ESBL-EAEC infection. **(A)** The relative abundance of top 20 bacterial genera in calf feces. **(B)** Top 50 bacteria of fecal samples presented using cluster heatmap analysis in diarrheal (D) or healthy (H) calves. In the graph of species clustering, the default species are UPGMA clustered according to the Pearson correlation coefficient matrix of their constituent data and arranged according to the clustering results. The red color block indicates that the abundance of the genus in this group is higher than in the other groups, while the blue color block indicates that the abundance of the genus in this group is lower than in the other groups. The corresponding relationship between the color gradient and the value is shown in the gradient color block.

**Figure 2:**
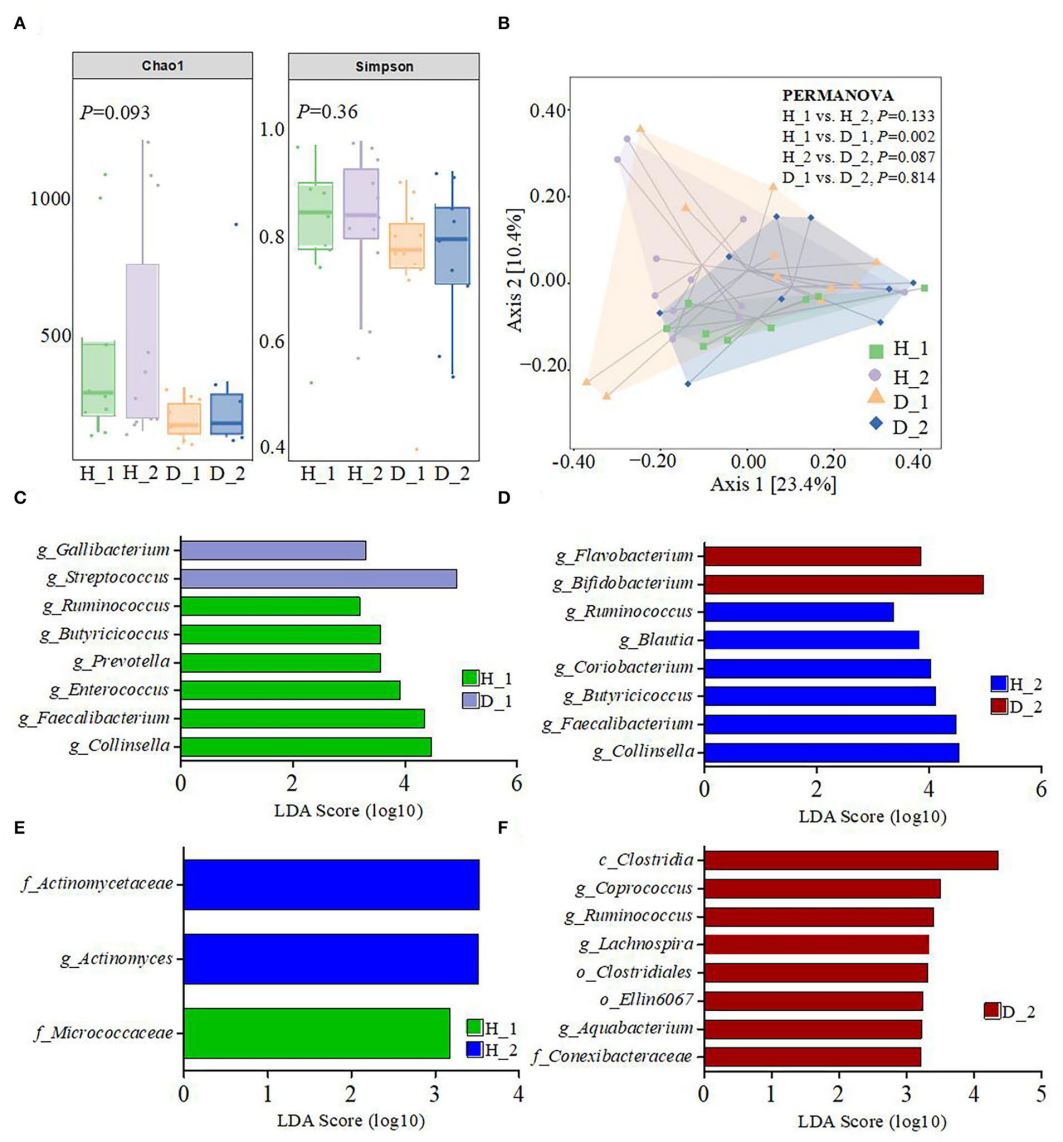
Gut microbiota diversity of neonatal calves post-ESBL-EAEC infection. **(A)** The α-diversity of different groups by Chao1 and Simpson indices. Data were presented as mean ± SEM values. *P-*values were determined using the nonparametric Kruskal–Wallis test. **(B)** Principal coordinate analysis (PCoA) of fecal bacteria was performed based on the weighted UniFrac distance matrix. The statistical tests were accomplished using PERMANOVA, with 999 permutations. The enriched gut microbiota taxa were shown by LEfSe [linear discriminant analysis (LDA) coupled with effect size measurements] of H_1 vs D_1 **(C)**, H_2 vs D_2 **(D)**, H_1 vs H_2 **(E)**, and D_1 vs D_2 **(F)**.

**Figure 3:**
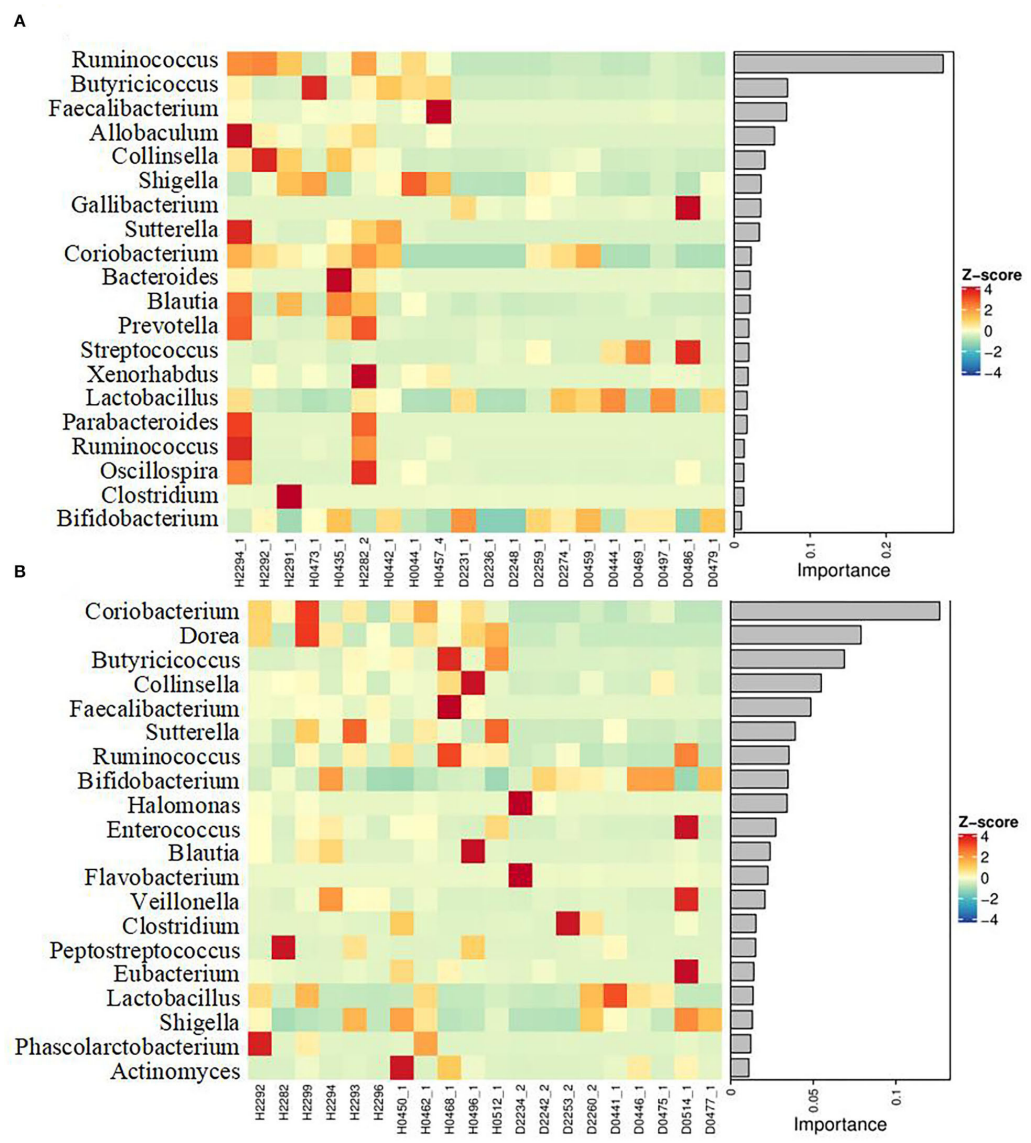
Differentiated genera were displayed using a random forest supervised machine learning algorithm between H_1 vs D_1 **(A)** and H_2 vs D_2 **(B)**. The respective name of the bacterial genus is shown on the left. The relative abundance of genera was clustered using a UPGMA dendrogram and showed in a heatmap. The color indicates the relative abundance of the genus in the group of samples, and the corresponding relationship between the color gradient and the value is shown in the gradient color block. The genus variation is shown using Z-Score. The top 29 genera in the fecal samples were included and the rank values are shown using the Importance Index.

To obtain more information regarding the impact of ESBL-EAEC infection on neonatal calves, the selected fecal samples were subjected to 16S rRNA gene sequencing to evaluate the diversity, community, and stability of fecal microbiota in different phases, including H_1, H_2, D_1, and D_2 (Supplementary Table 1). A total of 4,756,074 high-quality sequences were acquired from 40 fecal samples of 40 corresponding calves after quality control and filtration, and they were assigned to 7,766 ASVs based on a 99% nucleotide sequence similarity. A total of 10 bacterial phyla were shared among the four groups. Among these, Firmicutes, Actinobacteria, Proteobacteria, Bacteroidetes, and Fusobacteria accounted for a major proportion, while more members of Bacteroidetes and Fusobacteria were found in the healthy groups (Supplementary Figure 1A). Simultaneously, the relative abundance of *Coriobacteriaceae, Ruminococcaceae, Veillonellaceae, Bacteroidaceae*, and *Lachnospiraceae* was higher in the healthy groups, while the populations of *Bifidobacteriaceae, Lactobacillaceae*, and *Streptococcaceae* were enriched in the diarrheal group (Supplementary Figure 1B). Among them, remarkably abundant populations of *Collinsella, Prevotella, Ruminococcus, Faecalibacterium, Comamonas, Butyricicoccus, Blautia*, and *Oscillospira* were found in both H_1 and H_2 groups at the genus level (Figures 1A,B). In the diarrheal group, at 2–7 days, the relative abundance of *Escherichia-Shigella* tended to be lower, while it increased during 8–14 days of the D_2 phase. The relative abundance of almost all top 50 bacterial genera changed a lot over the whole period, depending on the affecting pathogen and the age of the animal. Besides, a decline in the microbial composition was observed in the D_1 and D_2 groups relative to H_1 and H_2 groups, as shown by Chao1 and Simpson indices (Figure 2A). The perturbations in microbial diversity and stability were further investigated via detecting beta diversity according to principal co-ordinate analysis (PCoA) based on weighted UniFrac distance. Indeed, the overall difference in the microbial structure of the diarrheal groups was distinct from that of the healthy ones, particularly the significantly changed distance between H_1 and D_1 groups (Figure 2B, *P* = 0.002). Furthermore, the linear discriminant analysis (LDA) effect size (LEfSe) algorithm ranked *Collinsella, Faecalibacterium, Enterococcus, Prevotella, Butyricicoccus*, and *Ruminococcus* as the main distinguished bacterial taxa in H_1 calves, with conspicuous *Streptococcus* and *Gallibacterium* in D_1 calves (Figure 2C). Similarly, *Collinsella, Faecalibacterium, Butyricicoccus, Coriobacterium, Blautia*, and *Ruminococcus* were identified as the differentiated bacteria in the H_2 group and *Bifidobacterium* and *Flavobacterium* in the D_2 group (Figure 2D). Besides, the differences between two healthy groups and two diarrheal groups were also detected. The results revealed Actinomycetaceae as the main distinguished taxa in H_2 calves and Micrococcaceae in H_1 calves (Figure 2E). Coprococcus, Ruminococcus, Lachnospira, and Aquabacterium were the differentiated bacteria in the D_2 group (Figure 2F). The random forest supervised machine learning algorithm was utilized to construct the model linking to the diarrheic prediction of neonatal female calves. A total of 29 genera were included in the differentiation of groups to establish the machine learning model, and this model showed an overall accuracy of 80% for predicting healthy and diarrheal gut microbiota. In the light of three-fold cross-validation, our results demonstrated that the same five bacteria, belonging to the 29 identified genera, did not change among the sampling days, including *Ruminococcus, Butyricicoccus, Faecalibacterium, Collinsella*, and *Coriobacterium*, which mostly resulted in the discrimination power of health status with improved performance (Figure 3). The Importance Index was used to plot the relative rank of relative abundances of five microbial markers, and the relative abundances of the five bacteria were found to be higher in the healthy groups. Briefly, these data demonstrated that ESBL-EAEC infection was closely related to the alteration in the structure of the gut microbial community, and the difference in ranks between these taxa suggested that *Gallibacterium, Flavobacterium, Bifidobacterium*, and *Streptococcus* were more ubiquitous in diarrheal gut microbiota, while *Ruminococcus, Butyricicoccus, Faecalibacterium, Collinsella*, and *Coriobacterium* were more prevalent microbial markers in healthy neonatal female calves relative to other taxa.

### Comparison of Temporal Changes in Fecal Metabolome Composition With the Growing Age and Specific Fecal Metabolites Mediating Diarrhea Resistance

**Figure 4:**
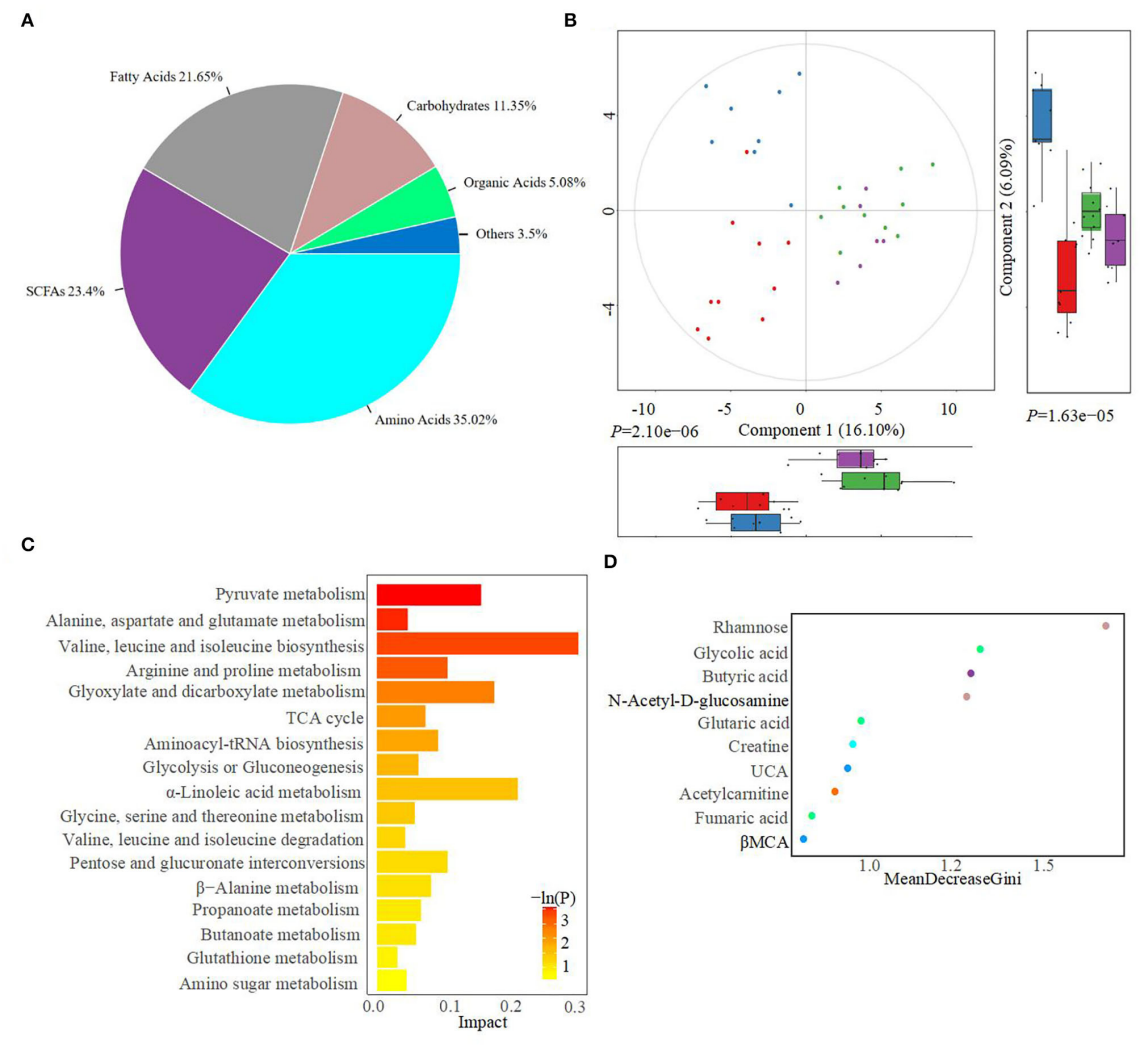
Alterations in the fecal metabolome profiles of neonatal calves post-ESBL-EAEC infection. **(A)** The classifications of total metabolome compounds in H_1, H_2, D_1, and D_2 groups. The total number of significantly changed metabolites in this class is indicated and the corresponding proportions are shown in parentheses. **(B)** Partial least squares discriminant analysis (PLS-DA) was used here to cluster the fecal metabolome profiles of calves. Metabolome profile for the H_1, H_2, D_1, or D_2 groups is shown in the same color, respectively. Data were presented as mean ± SEM values. *P*-values were acquired using the nonparametric Kruskal–Wallis test. **(C)** KEGG pathway enrichment analysis was associated with dramatically changed metabolites. The respective name of the KEGG pathway is shown on the left, and the corresponding *P*-value is shown on the right with a gradient color. *P-*values were acquired following two-side Fisher's exact tests with Benjamini-Hochberg correction for multiple testing. **(D)** Differentiated metabolites were displayed using a random forest supervised machine learning algorithms among H_1, H_2, D_1, and D_2 groups. The respective name of the metabolite is shown on the left. The top 10 metabolites in fecal samples are shown in different colors, and the rank values are shown as MeanDecreaseGini.

**Figure 5:**
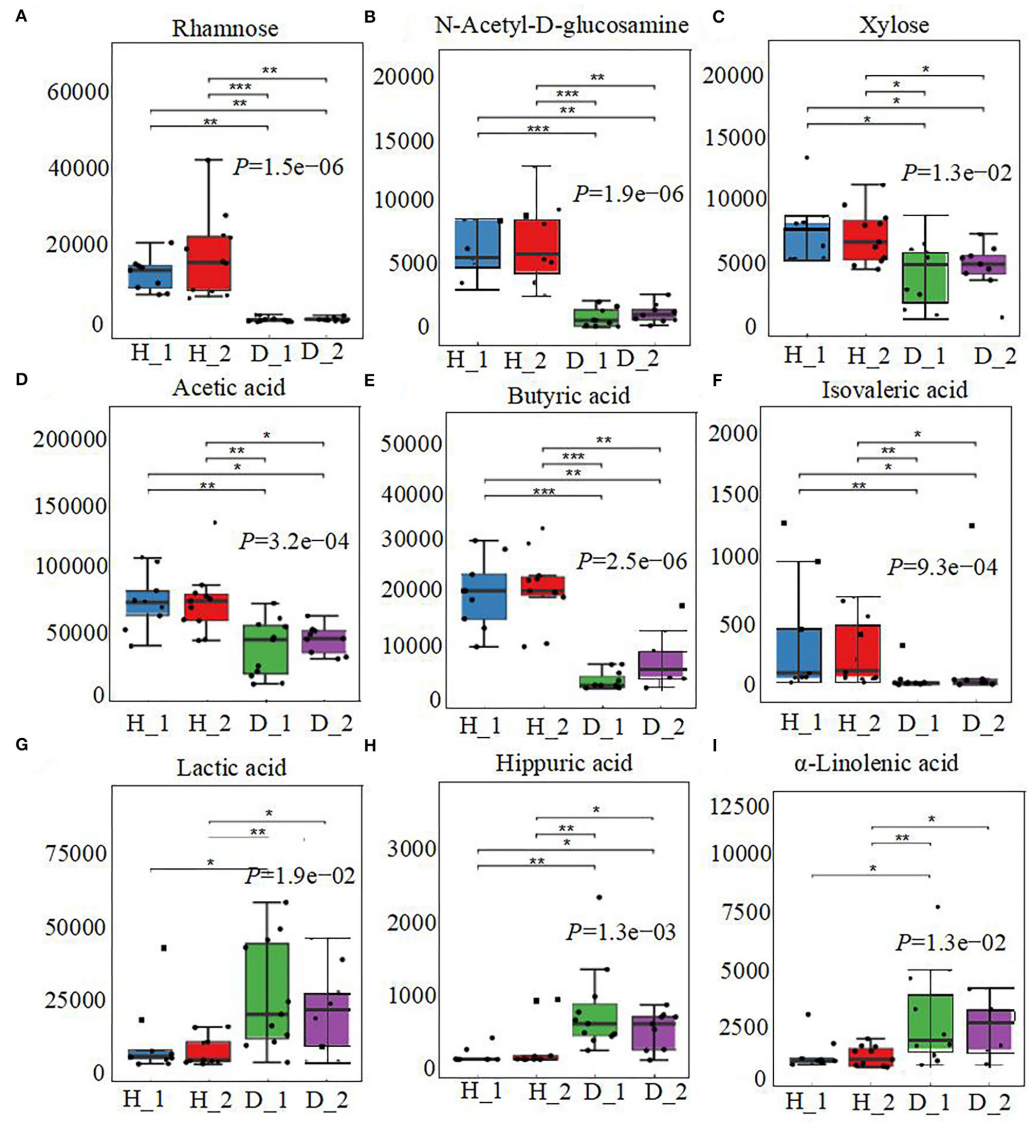
The alterations in fecal metabolome profiles of neonatal calves post-ESBL-EAEC infection. The concentrations of fecal rhamnose **(A)**, N-acetyl-D-glucosamine **(B)**, xylose **(C)**, acetic acid **(D)**, butyric acid **(E)**, isovaleric acid **(F)**, lactic acid **(G)**, hippuric acid **(H)**, and α-linolenic acid **(I)** are displayed as box and dot plots. Data were presented as mean ± SEM values. *P-*values were determined using the nonparametric Kruskal–Wallis test. *P ≤ 0.05, **P ≤ 0.01, ***P ≤ 0.001.

The fecal metabolome was further analyzed using untargeted metabonomics to obtain a systematic understanding of the interactions between intestinal epithelium, gut microorganisms, and their associated metabolites. Herein, fecal metabolites of healthy (*n* = 20) or diarrheal calves (*n* = 20) were analyzed with ultra-performance liquid chromatography coupled to tandem mass spectrometry (UPLC-MS/MS) system. Classification of different metabolites indicated that 35.02% of the compounds were amino acids, 23.4% were SCFAs, 21.65% were fatty acids, and 11.35% were carbohydrates (Figure 4A). Similarly, the healthy fecal metabolome separated widely from the diarrheal groups based on PLS-DA analysis, considering their allocated groups (Component 1, *P* = 2.10e−06; Component 2, *P* = 1.63e−05; Figure 4B). Unsurprisingly, dispersed data points on the plots of the metabolome were clearly displayed between H_1 and D_1, H_2 and D_2, H_1 and H_2, and D_1 and D_2 groups (Supplementary Figure 2). According to the markedly altered metabolites, enriched KEGG pathway analyses were involved with pyruvate metabolism, valine, leucine, and isoleucine biosynthesis, TCA cycle, glycolysis or gluconeogenesis, propanoate metabolism, and α-linoleic acid metabolism (Figure 4C). Importantly, the random forest supervised machine learning algorithm was also utilized to construct the model linking to diarrheic metabolome prediction of neonatal female calves. Herein, 10 most prominent fecal metabolites contributed to the discrimination power of health status in the dairy calf, including rhamnose, N-acetyl-D-glucosamine, and butyric acid. The relative rank of the relative abundance of these 10 metabolite biomarkers was plotted against the healthy status represented by the score of MeanDecreaseGini (Figure 4D). Among them, various unabsorbed carbohydrates were listed as potential biomarkers on comparison of the healthy calves with the diarrheal calves, which included rhamnose (*P* = 1.5e−06), N-acetyl-D-glucosamine (*P* = 1.9e−06), and xylose (*P* = 1.3e−02) (Figures 5A–C). The production of SCFAs correlated with colonic bacterial fermentation of unabsorbed carbohydrates and dietary fiber (Cummings et al., 1987). The relative production levels of fecal SCFAs, such as acetic acid (*P* = 3.2e−04), butyric acid (*P* = 2.5e−06), and isovaleric acid (*P* = 9.3e−04), were also compared in this study (Figures 5D–F). In addition, enriched concentrations of lactic acid (*P* = 1.9e−02), hippuric acid (*P* = 1.3e−03), and α-linolenic acid (*P* = 1.3e−02) were found in diarrheal groups (Figures 5G–I). Thus, the difference in the ranks of these metabolites and the corresponding production levels revealed that rhamnose, N-acetyl-D-glucosamine, xylose, acetic acid, butyric acid, and isovaleric acid were more prevalent in the healthy fecal metabolome of neonatal female calves, while lactic acid, hippuric acid, and α-linolenic acid were more prevalent in the diarrheal metabolome relative to other detected metabolites.

### Correlation Between Bacterial and Metabolite Markers in Disease Onset Prediction in Neonatal Intestine

**Figure 6:**
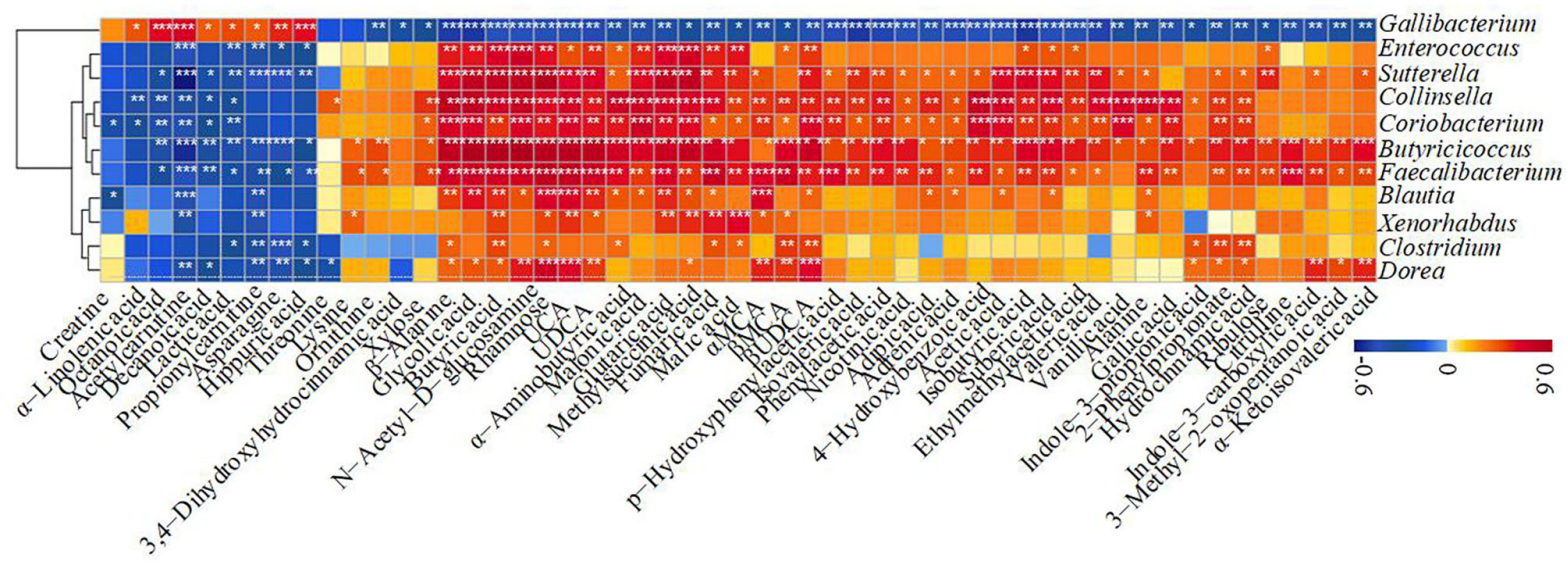
The Spearman correlation between differentiated gut microbial taxa and fecal metabolites in H_1 vs H_2 vs D_1 vs D_2 groups. Red squares indicate a positive correlation, and blue squares indicate a negative correlation. The intensity of the color was proportional to the strength of the Spearman correlation. *P ≤ 0.05, **P ≤ 0.01, ***P ≤ 0.001.

The correlation of significantly altered metabolites with specific differentiated microbial taxa was revealed directly. Spearman rank correlation analysis indicated a strong positive correlation between abundant rhamnose and *Sutterella* (*R* > 0.74, *P* = 5.4e−08), *Butyricicoccus* (*R* > 0.71, *P* = 3.2e−07), *Faecalibacterium* (*R* > 0.68, *P* = 1.4e−06), *Dorea* (*R* > 0.60, *P* = 4.0e−05), *Collinsella* (*R* > 0.51, *P* = 0.00081), and *Coriobacterium* (*R* > 0.48, *P* = 0.0014) in H_1 and H_2 groups (Figure 6). Similarly, a significantly positive correlation between abundant N-acetyl-D-glucosamine and *Butyricicoccus* (*R* > 0.77, *P* = 6.3e−09), *Sutterella* (*R* > 0.68, *P* = 1.6e−06), *Collinsella* (*R* > 0.61, *P* = 4.0e−05), *Faecalibacterium* (*R* > 0.59, *P* = 5.8e−05), *Coriobacterium* (*R* > 0.56, *P* = 0.00018), and *Dorea* (*R* > 0.48, *P* = 0.0018) (Figure 6) was noticed. Notably, *Butyricicoccus* also showed the strongest correlation with enriched butyric acid (*R* > 0.75, *P* = 3.2e−08), isobutyric acid (*R* > 0.55, *P* = 0.00022), isovaleric acid (*R* > 0.46, *P* = 0.0031), glycolic acid (*R* > 0.75, *P* = 1.8e−08), and UDCA (*R* > 0.69, *P* = 1.0e−06), while *Collinsella* exhibited the strongest correlation with enriched acetic acid (*R* > 0.56, *P* = 0.00022). The microbes mentioned above were also strongly linked to other unabsorbed carbohydrates, SCFAs, bile acids, and indole upregulations, and negatively related to α-linolenic acid, hippuric acid, lactic acid, and amino acid consumption. Also, it should be noted that there existed a strong correlation between *Gallibacterium* and hippuric acid (*R* > 0.50, *P* = 0.00099) and lactic acid (*R* > 0.40, *P* = 0.01). Thus, massive reduction in the unabsorbed carbohydrates, SCFAs, and some other prebiotics was probably due to the decrease in the abundance of *Sutterella, Butyricicoccus, Faecalibacterium, Dorea, Collinsella*, and *Coriobacterium* observed in healthy calves, while increased hippuric acid and lactic acid content related to affluent *Gallibacterium*. These observations were similar to the obtained microbial and metabolite biomarkers. Thus, a decline in some of the commensal bacteria caused by ESBL-EAEC infection was apparently linked to a drop in fecal unabsorbed carbohydrates and derived SCFA production (including acetic acid, butyric acid, isobutyric acid, and isovaleric acid), thus inducing temporal destruction of intestinal homeostasis. Of note, our findings suggested that unabsorbed carbohydrates or early dietary fiber administration could ameliorate the intestinal health status of neonatal female calves.

## Discussion

Calves are a group of animals that are highly susceptible to various enteric infections considering the immature immune system and gastrointestinal tract. According to statistics on the morbidity of 450,000 heifers of China, our research group concluded that the morbidity of suckling calves accounted for 51.4% of the cases, of which the incidence of calf diarrhea accounted for 72.8% of the cases (White Paper on China Dairy Replacement 2020). Hence, prevention of calf diarrhea and timely intervention still pose a great challenge. The prevalent diarrheagenic *Escherichia coli* (DEC) causes an aggravation of the sporadic clinical diarrhea cases, resulting in the outbreak of gastroenteritis around the world (Schultsz et al., 2000). They can be categorized into six pathotypes: enteropathogenic *E. coli* (EPEC), enterohaemorrhagic (Shiga-toxin producing) *E. coli* (EHEC/STEC), enterotoxigenic *E. coli* (ETEC), enteroaggregative *E. coli* (EAEC), enteroinvasive *E. coli* (EIEC), and diffusely adherent *E. coli* (DAEC) (Kaper et al., 2004). Even worse, the pathogenesis of ESBL-EAEC remains unclear, and antibiotic abuse accelerates the rapid spread of MDR, which poses a severe threat to public health (Boucher et al., 2009). Multi-drug resistant ESBL-EAEC infection correlates with the extensive clinical diarrheal cases among animals and humans, particularly the rising occurrence of extended-spectrum beta-lactamase (ESBL)-producing isolates (Valat et al., 2014). Here, we aimed to attenuate the suffering associated with the post-ESBL-EAEC infection among neonatal calves by investigating predictive biomarkers with the aim of blocking further dissemination of resistance. We directly isolated clinical ESBL-EAEC isolates from neonatal calves in the conventional pasture, which had been proved to harbor MDR genes and enterotoxin EAST1. ESBL-EAEC strains with highly expressed EAST1 have been proved to cause diarrhea principally in humans, and later facilitate rapid adaption and propagation of bacteria in calves, piglets, and the other animals (Menard and Dubreuil, 2002). In most cases, ESBL-EAEC infections represent asymptomatic carriage and are self-limiting in the host, but some external factors, such as colostrum, diet, and environmental microbial community, correlate closely leading to the development and progression of calf diarrhea (Cho and Yoon, 2014). Currently, we only separated EAEC strains due to geographical restrictions and limited large ranch opening permissions. So, the data here could only reveal the antibacterial effects of the host on ESBL-EAEC. Nevertheless, we will surely proceed with further investigations on the regulatory role of the host in the pathogenesis of other DEC strains thoroughly in our subsequent clinical studies, along with the isolation of these strains from the calves of other conventional pastures in the other parts of China, thus systematically clarifying the systematic mechanism of DEC intervention action on gut health and development during the early days of life.

Alterations in the gut microbial colonization in the early life often contribute to longstanding effects on rumen microbes and host phenotype (Furman et al., 2020). In the current study, the abundance of nearly all top 50 bacterial genera changed a lot from 2–7 days to 8–14 days of age. Such findings were similar to the shift in the hindgut microbiota in healthy dairy calves during the first 6 weeks of age (Song et al., 2018). Diet composition correlates with the alterations in the gut microbial diversity in these young calves (Dill-McFarland et al., 2017), and the detected changes in the hindgut microbiota of healthy calves correlate with colostrum feeding, milk replacer components, and calf starter feeding (Song et al., 2019). To avoid these limitations, the calves in this trial were fed with the same batch of heated colostrum (60°C, 60 min) and milk replacer (same amount under the same feeding phase), to facilitate the detection of the main host factors involved in mediating diarrhea resistance against ESBL-EAEC in neonatal female calves. Although the temporal changes in gut microbiota between H_1 and H_2 groups or D_1 and D_2 groups were similar, and the relative abundance of total bacterial genera showed no obvious difference among the groups, a significant tendency toward higher Chao1 and Simpson indices and dispersed bacterial community in healthy groups than diarrheal groups revealed that perturbations owing to ESBL-EAEC infection resulted in the fluctuation of the colonization of commensal bacteria and microbial dysbiosis. In our study, no calves received therapeutic antimicrobials or medical treatments, thus avoiding severe negative impact on the early development of neonatal microbial diversity and stability over the first 2 weeks of life and reflecting the real self-regulation ability of the host.

To gain insight into the alteration of fecal microbiome post-ESBL-EAEC infection, taxonomic and bacterial compositions of all fecal samples were compared immediately. Similar to the reports of a previous publication (Ma et al., 2020), most of the diarrheal cases occurred in female calves from 4 to 10 days. Various species of *Escherichia-Shigella* are widely known as the major enteric pathogens and cause calf diarrhea (Bartels et al., 2010). In this study, a lower relative abundance of *Escherichia-Shigella* was observed at 2–7 days in the D_1 than in H_1 groups, while a higher abundance was noticed at 8–14 days in the D_2 than in H_2 groups, indicating the gradual colonization and a favorable position of ESBL-EAEC in the gut lumen post-infection. The detected higher abundance of *Escherichia-Shigella* during the first week suggested that calves were more susceptible to infections considering their exposure to more number of opportunistic pathogens during this period (Song et al., 2018). Importantly, gut commensal bacteria are closely linked to immune recognition, host nutrient acquisition, and pathogen exclusion, thus linking endogenous and exogenous factors (Littman and Pamer, 2011; Clemente et al., 2012). Our data demonstrated that ESBL-EAEC infection promoted the diarrheal process by altering the composition of gut microbiota, which included a severe reduction in the abundance of *Sutterella, Collinsella, Prevotella, Faecalibacterium, Butyricicoccus, Ruminococcus, Blautia*, and *Oscillospira*. A higher prevalence of mucin- or SCFA-producing bacteria has been reported in the colon or fecal microbiota in healthy calves (Sokol et al., 2009; Lee et al., 2013; Graziani et al., 2016; Zhou et al., 2018; Vacca et al., 2020). However, the interference of those commensal bacteria colonizations correlated with diarrhea in animals (AlShawaqfeh et al., 2017). Besides, the enriched antibiotic resistance genes detected in diarrheal groups reflect the potential hazard of increased gene propagation and the risk of failure of future antimicrobial treatment. In view of the negative effect of the perturbation on the temporal development of microbial community due to ESBL-EAEC infection, a timely prediction of diarrhea based on microbial and metabolite biomarkers could be a promising approach to avoid the occurrence of such perturbations. LEfSe analysis of H_1 vs D_1 and H_2 vs D_2 groups highlighted that *Ruminococcus, Butyricicoccus, Faecalibacterium, Collinsella*, and *Coriobacterium* could be deemed as the indicator phylotypes of active intestinal tract. A similar change in the gut microbiota has been validated in patients with inflammatory bowel disease (IBD) (Pittayanon et al., 2020). However, diarrheal calves were associated with prevalent *Gallibacterium, Flavobacterium, Bifidobacterium*, and *Streptococcus*. Notably, the microbial community of the hindgut was dominated by lactic acid bacteria, including *Lactobacillus, Streptococcus*, and *Bifidobacterium*, as indicated by abundant lactic acid in the diarrheal groups, which can be attributed to the prominent contribution of hindgut fermentation in metabolizable energy supply during the first weeks after birth (Castro et al., 2016). Genus *Bifidobacterium* is known to prevail in the neonatal gut, especially when the colostrum is fed during the first 12 h of life (Malmuthuge et al., 2015). In our study, the relative abundance of digesta-associated *Bifidobacterium* was higher during 2–7 days than during 8–14 days, indicating that the consumption of milk oligosaccharides could result in a higher abundance of this genus (Lozupone et al., 2013). ESBL-EAEC infection could markedly increase the *Bifidobacterium* population, and also enrich *Lactobacillus* and *Streptococcus* in the diarrheal groups. Based on these findings, the results demonstrated that the hindgut microbiota of the neonatal female calves was similar to the microbial composition of monogastric animals, harboring the capabilities of adaption to an anaerobic environment and utilization of available substrates to construct their colonization niches. *Gallibacterium* is associated with a wide range of pathological changes in poultry (Persson and Bojesen, 2015), thus indicating the potential risks of intestinal infections in neonatal calves. *Flavobacterium* is detected to be a common species among the bacterial communities in diarrhea-affected cattle (Ateba et al., 2021). Further studies are needed to probe into the mutual influence of these microbial markers on improved gut health in young ruminants.

As for metabolome data and modeling algorithms of metabolites, various unabsorbed carbohydrates were listed as potential biomarkers, including rhamnose, N-acetyl-D-glucosamine, and xylose, accompanied by enriched acetic acid, butyric acid, and isovaleric acid. Increased amounts of lactic acid, hippuric acid, and α-linolenic acid were observed in the diarrheal groups, which highlighted that the differences in the fecal production of these nine metabolite markers closely reflect their absolute difference between “healthy” and “diarrheal” metabolomes. Plunged commensal bacteria, such as *Sutterella, Butyricicoccus, Faecalibacterium, Dorea, Collinsella*, and *Coriobacterium*, strongly correlated with the reduced unabsorbed carbohydrates, SCFAs, and bile acids, and indole upregulation, and negatively related to α-linolenic acid, hippuric acid, lactic acid, and amino acid consumption over the whole time period, underlining the importance of both improved microbiota and medicinal benefits of their derived metabolome on the ESBL-EAEC infection state. There was also a strong correlation between *Gallibacterium* and hippuric acid and lactic acid. Thus, a simple prediction model using gut microbial markers was still challenging and limited to predict diseases of humans and animals. Early-life hindgut microbiota and fecal metabolome analyses should be combined for the accurate prediction of the diarrheal processes (such as ESBL-EAEC infection) in young calves.

Other kinds of metabolites were also differentiated in our study, including UDCA and indoles. UDCA, a natural secondary bile acid derived from gut microbiota, is discovered to possess an excellent effect on colonic epithelial cell protection against oxidative damage and cell apoptosis (Barrasa et al., 2013). Indoles belong to gut microbiota-derived tryptophan metabolites, which could influence inflammatory responses (Krishnan et al., 2018). Importantly, the beneficial effects of SCFA on the gut mucosal immune response also provided adjunctive effects in the fight against ESBL-EAEC infection (Hiltz and Laarman, 2019; Liu et al., 2021). These aforementioned metabolites could also display prebiotic properties against ESBL-EAEC infection. In our future research, we would explore the direct role of these above-mentioned commensals (*Butyricicoccus, Faecalibacterium, Ruminococcus, Collinsella*, and *Coriobacterium*) in mediating the metabolism of unabsorbed carbohydrates, SCFA, and other prebiotics and antibacterial effects using culture technique, proteomics technique, and targeted metabolomics. It can be achieved by directly comparing ESBL-EAEC-infected diarrheal neonatal calves with those purely isolated from the hindgut bacteria of healthy calves. Linoleic acid, a dietary polyunsaturated fatty acid (PUFA), can serve as a key biomarker for the progression of ulcerative colitis and gut microbiota dysbiosis, and destroy the cell membrane of some probiotics (Lv et al., 2020; Tang et al., 2020), which is consistent with enriched linoleic acid metabolism pathway in our KEGG analysis. In addition, hippuric acid, a protein-bound uremic toxin, correlated with the upregulation of pro-inflammatory cytokines and oxidative stress, which could accelerate the deterioration of disease and indicated its utility in calf feces as a plausible hallmark of frailty post-ESBL-EAEC infection (Watanabe et al., 2011). Future research exploring the direct relationship between *Gallibacterium* and hippuric acid and lactic acid metabolisms was also needed. Our limitations were that limited sampling time points (2–14 days) were available, which could not correctly reflect the dynamic changes of the microbiota and metabolites during different infection stages, and the inherent influential mechanisms remained elusive. The sampling number is limited to assess the accuracy of the current model and its broad application to different herds. Therefore, further research is needed to determine the exact role of these biomarkers in the progression of calf diarrhea with much more sampling time points and adequate populations to detect their prediction specificity of diarrhea induced by ESBL-EAEC infection.

The successful colonization of hindgut microbiota and increased microbial diversity and stability are vital features for optimizing the better performance of neonatal calves. ESBL-EAEC infection in the early days led to drastic changes in the hindgut microbial community and altered fecal metabolites especially during the first 2 weeks of age, suggesting that early infections by these bacteria could probably have a negative impact on the long-term health of female calves. Previous studies clearly indicated the early life stage as a critical window for gut microbiota manipulation to mediate the metabolome and immunity of neonatal calves. Thus, future studies concerning the impact of early control of pathogens and supplementation with unabsorbed carbohydrates or dietary fiber on gut health and productivity of calves are urgently needed, utilizing multi-omics analyses to elucidate the effect of the interaction between these biomarkers on the gut health.

## Conclusion

Collectively, multi-omics analyses of fecal samples of neonatal calves indicated the differences in the hindgut microbiota and fecal metabolites. Using the random forest model and Spearman correlation analysis, the data provided innovative insights into the exact predictions of diarrhea induced by ESBL-EAEC using commensals and associated unabsorbed carbohydrates among neonatal calves. In addition, the results highlighted the possibilities of employing hindgut microbiota and associated metabolites for predicting many other intestinal infections or diseases in neonatal food-producing animals, thus facilitating the reduction of antimicrobial usage.

## Data Availability Statement

The datasets presented in this study can be found in online repositories. The names of the repository/repositories and accession number(s) can be found in the article/Supplementary Material.

## Ethics Statement

The animal study was reviewed and approved by the Institutional Ethics Committees of China Agricultural University.

## Author Contributions

ZC, ZH, YM, YW, WW, HY, and SLi designed the experiments. YM and ZH conducted the experiments and analyzed the data. SY, YM, SZ, SLiu, XC, and JX collected the samples and performed the analysis of the samples. ZC and ZH wrote the manuscript. All authors read and approved the final manuscript.

## Funding

The present research was supported by grants from the National Key Research and Development Program of China, Grant Number: 2021YFF1000703-03.

## Conflict of Interest

The authors declare that the research was conducted in the absence of any commercial or financial relationships that could be construed as a potential conflict of interest.

## Publisher's Note

All claims expressed in this article are solely those of the authors and do not necessarily represent those of their affiliated organizations, or those of the publisher, the editors and the reviewers. Any product that may be evaluated in this article, or claim that may be made by its manufacturer, is not guaranteed or endorsed by the publisher.

## Supplementary Material

Supplementary Table 1 Sampling information of calves used in the current study.

Supplementary Table 2 Genomic analysis of 1587 strain.

Supplementary Table 3 Antibiotic susceptibility analysis of 1587 strain.

Supplementary Figure 1 Gut microbiota assembly of neonatal calves post-ESBL-EAEC infection. The relative abundance of fecal bacterial phylum **(A)** and family **(B)** represented 99.5% of the community.

Supplementary Figure 2 Cognate metabolomics analyses of fecal samples in healthy and diarrheic calves. Partial least squares discriminant analyses (PLS-DA) for neonatal calves in H_1 vs D_1 **(A)**, H_2 vs D_2 **(B)**, H_1 vs H_2 **(C)**, and D_1 vs D_2 **(D)**. H, healthy calves; D, diarrheic calves.
