# Supplementary figures and images for: Temporal Changes in Fecal Unabsorbed Carbohydrates Relative to Perturbations in Gut Microbiome of Neonatal Calves: Emerging of Diarrhea Induced by Extended-Spectrum β-lactamase-Producing Enteroaggregative Escherichia coli
Source: Front Microbiol. 2022 Jul 7;13:883090. doi: 10.3389/fmicb.2022.883090 (PMC9301005; doi:10.3389/fmicb.2022.883090)

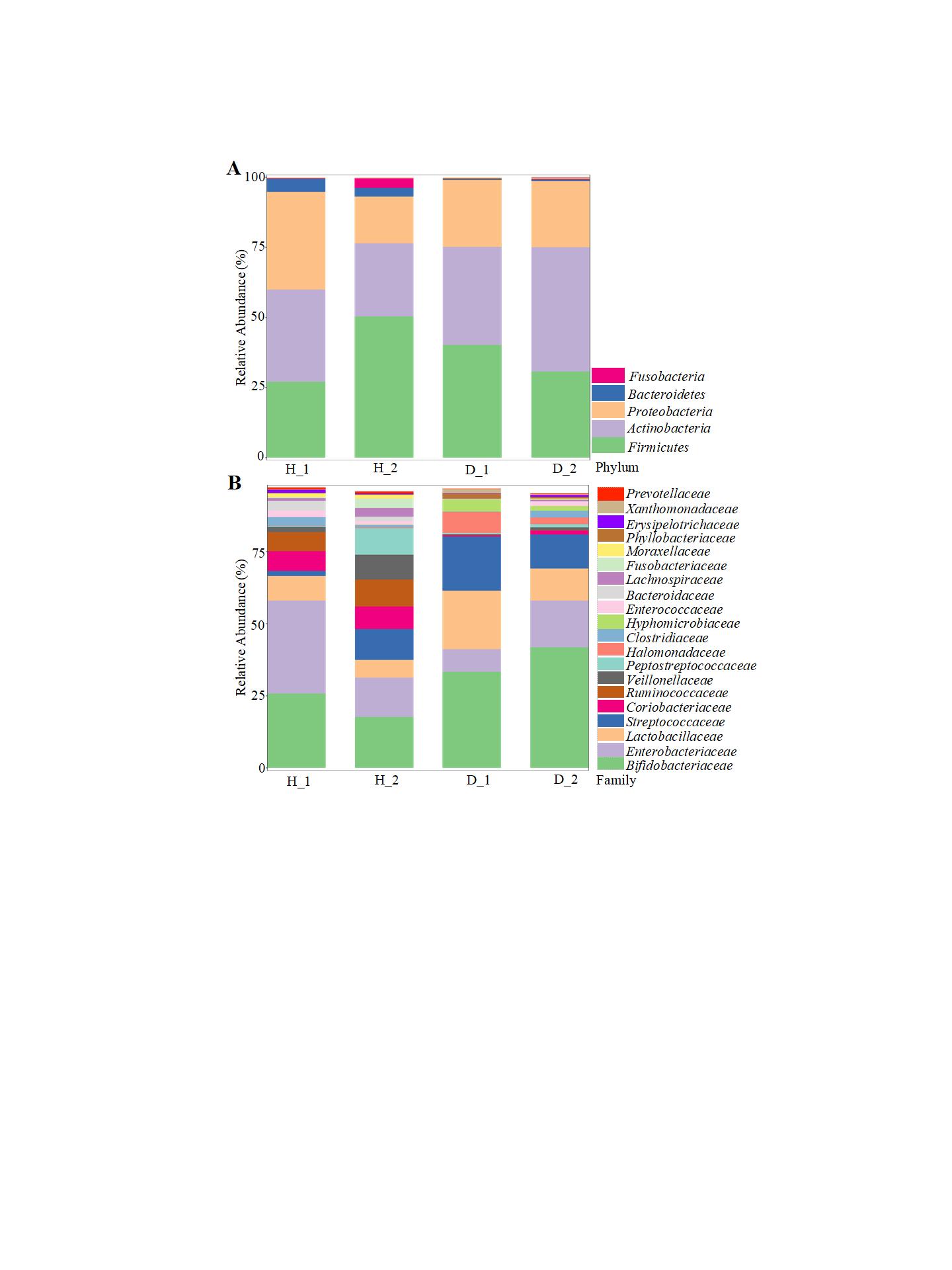

Supplement: Supplementary Figure 1 — Gut microbiota assembly of neonatal calves post-ESBL-EAEC infection. The relative abundance of fecal bacterial phylum (A) and family (B) represented 99.5% of the community. [file Image_1.JPEG]

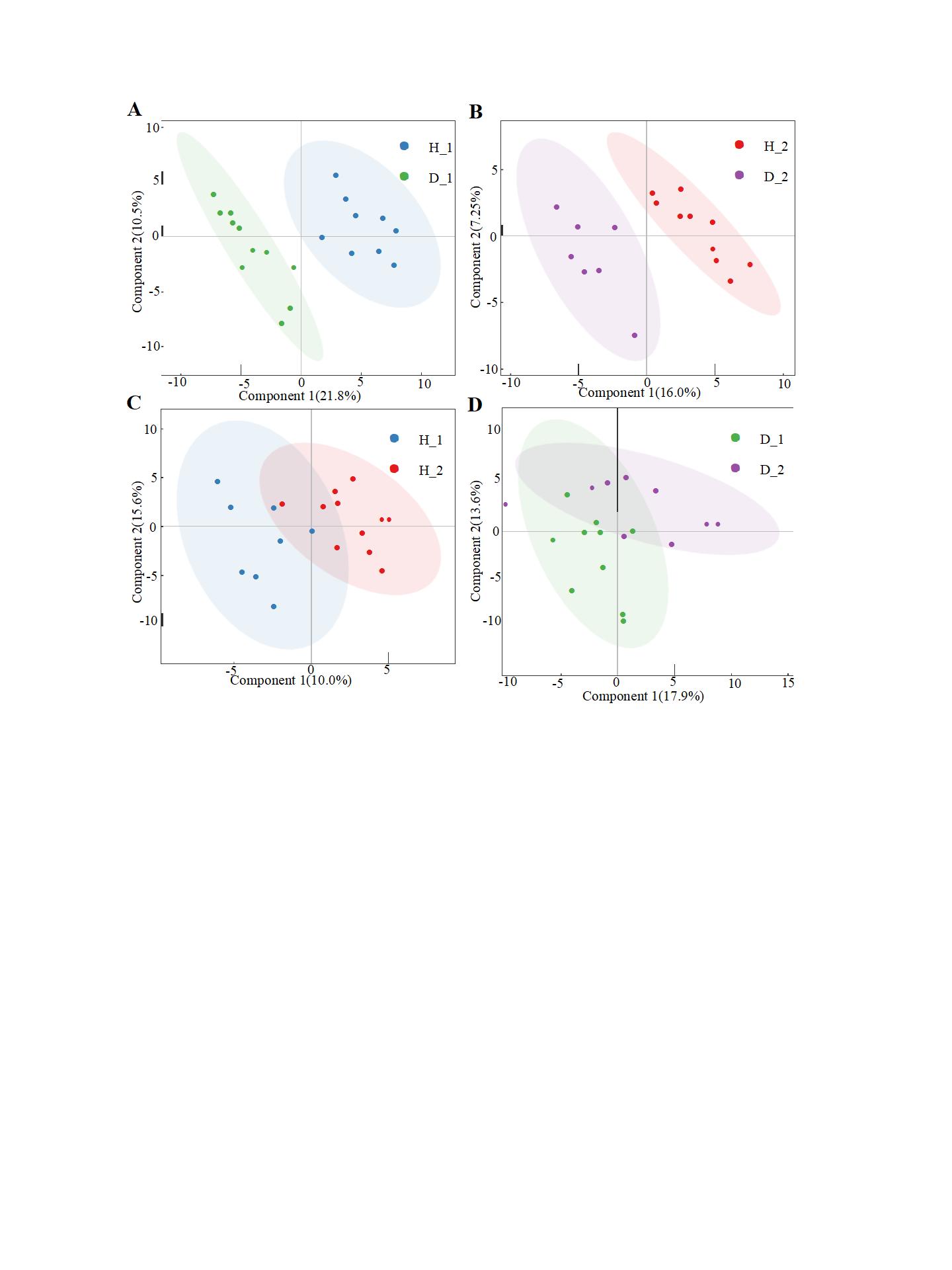

Supplement: Supplementary Figure 2 — Cognate metabolomics analyses of fecal samples in healthy and diarrheic calves. Partial least squares discriminant analyses (PLS-DA) for neonatal calves in H_1 vs D_1 (A), H_2 vs D_2 (B), H_1 vs H_2 (C), and D_1 vs D_2 (D). H, healthy calves; D, diarrheic calves. [file Image_2.JPEG]
